# Microbiological Quality of Reverse Osmosis Water of a Dialysis Unit: Analysis From a Tertiary Care Hospital in Imphal, India

**DOI:** 10.7759/cureus.80791

**Published:** 2025-03-18

**Authors:** Yendrembam Bidyalakshmi Devi, Subhrajit Bhattacharjee, Pranab Bhaumik, Kshetrimayum Mamta Devi, Anindita Aich, Sushan Subba

**Affiliations:** 1 Department of Microbiology, Regional Institute of Medical Sciences, Imphal, IND

**Keywords:** acinetobacter spp, dialysate, hemodialysis, microbial profile, water quality

## Abstract

Background

Patients undergoing maintenance hemodialysis (HD) are exposed to large volumes of treated water, making its microbiological quality crucial for patient safety. Contaminated dialysis water can introduce microbial pathogens into the bloodstream, increasing the risk of pyrogenic reactions, sepsis, and chronic inflammation. Water quality monitoring remains a challenge in many healthcare settings despite its importance. This study evaluates the microbiological quality of treated water and dialysate used in a tertiary care hospital dialysis unit in Imphal, India.

Methods

This observational study was conducted at the Regional Institute of Medical Sciences, Imphal, from August 2021 to January 2025. A total of 84 water samples were collected from different points in the dialysis system, including product water, storage water, middle port water, and return water. Samples were processed using membrane filtration and analyzed for microbial contamination using culture-based methods and Vitek 2 Compact for species identification. The findings were assessed against the Association for the Advancement of Medical Instrumentation (AAMI) standards for dialysis water quality.

Results

Microbiological analysis revealed varying contamination levels across different water sources. Product water exhibited the highest microbial presence, with *Acinetobacter* species being the most frequently detected organism. The most recent sample (January 2025) showed *Aeromonas* spp. and *Acinetobacter* spp. in product water. Some samples exceeded AAMI standards, with colony-forming unit (CFU) counts ranging from 104 to 274 CFU/100 mL in product water. Storage, middle port, and return water showed lower contamination levels. Out of the total 21 samples collected, unsatisfactory microbial growth was observed in three samples of product water and one sample of storage water as per AAMI standard, whereas no unsatisfactory growth was detected in any of the middle port water or return water samples. *Acinetobacter* *baumannii* was detected in all four water types, while other multidrug-resistant organisms, such as *Klebsiella pneumoniae*, were found in return water.

Conclusion

The study highlights fluctuating levels of microbial contamination in dialysis water, with *Acinetobacter* species being the predominant contaminant. Regular monitoring, stringent adherence to water treatment protocols, and strict infection control measures are essential to ensure dialysis water safety and improve patient outcomes.

## Introduction

The global burden of end-stage kidney disease (ESKD) is increasing, with approximately 100,000 new patients in India requiring renal replacement therapy each year [1-3]. Due to the limited availability of kidney donors and financial challenges, a significant number of patients in India rely on maintenance hemodialysis (HD) [4]. Patients undergoing maintenance HD are exposed to large volumes of water, ranging from 350 to 500 liters per week [5,6]. The treated water and dialysate used during dialysis come into direct contact with the patient’s blood. A thin membrane within the dialyzer separates the water from the blood, limiting the transfer of contaminants primarily based on their size. Although the usual movement of water during HD is from the blood to the dialysate, back-filtration may occur, allowing water to flow from the dialysate into the blood [7]. This process can introduce microbial contaminants into the bloodstream, potentially causing pyrogenic reactions, sepsis, and severe complications such as hypotension and shock, further worsening the patient's clinical condition [8].

Contaminated water used in dialysis can lead to the formation of biofilms within the HD system, releasing endotoxins. Once a biofilm develops, it becomes difficult to remove, even with regular disinfection, serving as a persistent source of endotoxins, peptidoglycans, and bacterial DNA fragments. These contaminants can cross the dialyzer membrane, triggering cytokine production and raising acute-phase reactants. Such interactions may result in acute intradialytic complications, including fever, chills, and hypotension [9]. Studies also suggest potential associations between water contamination and chronic health issues, such as β2-microglobulin amyloidosis and atherosclerosis [10,11].

Despite these risks, the microbial quality of dialysis water often remains overlooked. Ensuring high bacteriological quality of dialysis fluids is crucial, as better water quality can significantly improve patient outcomes and may even enhance survival rates [12,13]. Thus, this study was conducted to evaluate the bacteriological quality of treated water and dialysate used in the HD unit of a tertiary care center in Imphal, Manipur, India.

## Materials and methods

This observational study was conducted at the Regional Institute of Medical Sciences (RIMS), Imphal, from August 2021 to January 2025, in the Bacteriology Laboratory of the Department of Microbiology. Water samples were obtained from the Dialysis Unit of RIMS, Imphal. A total of 84 samples were analyzed during the study period, comprising 21 each of product water, storage water, middle port water, and return water samples.

Water samples were collected from the treated water system immediately downstream of the water purification system (reverse osmosis) tank and from the RO lines supplying the HD unit. Prior to sample collection, the sample ports were disinfected with alcohol and allowed to air dry. At each collection point, the valve was opened, and water was allowed to flow for at least two minutes at normal pressure and flow rate to ensure representative sampling. Using sterile gloves and the "clean catch" technique to minimize contamination, four types of samples were obtained: product water from the RO system, water from the machine storage tank, water from the middle port, and return water from the dialysis unit [14].

The collected water samples were appropriately labeled with details, including the source, machine type, date, and time of collection. Samples were transported carefully by holding the sterile bottle at the base and delivered to the laboratory promptly for analysis within one to two hours of collection. The samples were processed using the membrane filtration technique. During this process, the bottle was opened with one hand and tilted with the other, allowing the water to pass through a sterilized nylon membrane filter paper (Nexflo Membrane Filters, Moxcare Products Inc., India). These filter papers were incubated at 37°C, and bacterial colony formation was observed after 18-24 hours. Colonies from plates with positive growth were counted and expressed as colony-forming units (CFU) per milliliter. Gram staining was performed on the isolates, and growth on MacConkey agar was further confirmed using the Vitek 2 Compact (BIOMÉRIEUX, France). This process was consistently repeated for all four types of samples to ensure reliable monitoring [14].

For the interpretation of product water, storage water, middle port water, and return water, the colony count per milliliter (CFU/mL) was determined whenever bacterial growth was observed. The colony count was determined by multiplying the observed number of colonies by 100 and dividing by the volume of water filtered [14]. The maximum permissible levels for various water purity grades are summarized in Table 1 [14].

**Table 1:** Maximum levels of the different water purity grades AAMI: Association for the Advancement of Medical Instrumentation, CFU: colony-forming units, HD: hemodialysis

Maximum levels	AAMI water	European Pharmacopoeia		
		Regular water	Ultrapure water	HD fluid for infuset
Microbial contamination (CFU/mL)	200	<100	<0.1	<0.000001
Bacterial endotoxins (IU/mL)	<2	<0.25	<0.03	<0.03

## Results

The microbiological analysis of water samples collected over a period from August 2021 to January 2025 revealed varying distributions of microorganisms across different water sources, including product water, storage water, middle port water, and return water (Table 2). *Acinetobacter *spp. was the most frequently detected microorganism, appearing in multiple samples across different collection periods, particularly in product and return water. The most recent positive sample, collected in January 2025, revealed the presence of *Aeromonas *spp. and *Acinetobacter *spp. in product water. Several collection months, including December 2021, May 2022, September 2022, November 2022, March 2023, October 2023, January 2024, February 2024, and November 2024, showed no microbial growth across all water sources.

**Table 2:** Distribution of microorganisms detected according to source water NG: No growth; GNCB: Gram-negative coccobacilli; ASB: Aerobic spore-bearing bacilli; numbers in brackets depict the CFU/100 ml

Sample no.	Month/year of collection	Product water	Storage water	Middle port water	Return water
1	08/21	*Acinetobacter* spp. (230)	Acinetobacter spp. (20)	NG	NG
2	09/21	*Cupriavidus* spp. (120)	*Acinetobacter* spp. (100)	*Acinetobacter* spp. (126)	*Acinetobacter* spp. (132)
3	10/21	NG	NG	NG	*Acinetobacter* spp. (116)
4	11/21	*Acinetobacter* spp. (146)	*Acinetobacter* spp. (38)	NG	NG
5	12/21	NG	NG	NG	NG
6	01/22	GNCB (250)	NG	NG	NG
7	05/22	NG	NG	NG	NG
8	09/22	NG	NG	NG	NG
9	11/22	NG	NG	NG	NG
10	03/23	NG	NG	NG	NG
11	08/23	*Staphylococcus* spp. (130)	*Enterobacter* spp. (128)	*Enterobacter* spp. (56)	NG
12	09/23	*Staphylococcus* spp. (104)	NG	ASB (88)	NG
13	10/23	NG	NG	NG	NG
14	11/23	*Acinetobacter* spp. (114)	NG	NG	NG
15	12/23	*Pseudomonas* spp. (144)	*Comamonas* spp. (210)	*Stenotrophomonas* spp. (48)	*Klebsiella* spp. (30)
16	01/24	NG	NG	NG	NG
17	02/24	NG	NG	NG	NG
18	05/24	NG	*Chryseobacterium* spp. (120)	NG	NG
19	07/24	*Acinetobacter* spp. (134), *Ralstonia* spp. (44)	NG	NG	*Acinetobacter* spp. (58)
20	11/24	NG	NG	NG	NG
21	01/25	*Aeromonas* spp. (226), *Acinetobacter* spp. (48)	NG	NG	NG

**Figure 1:**
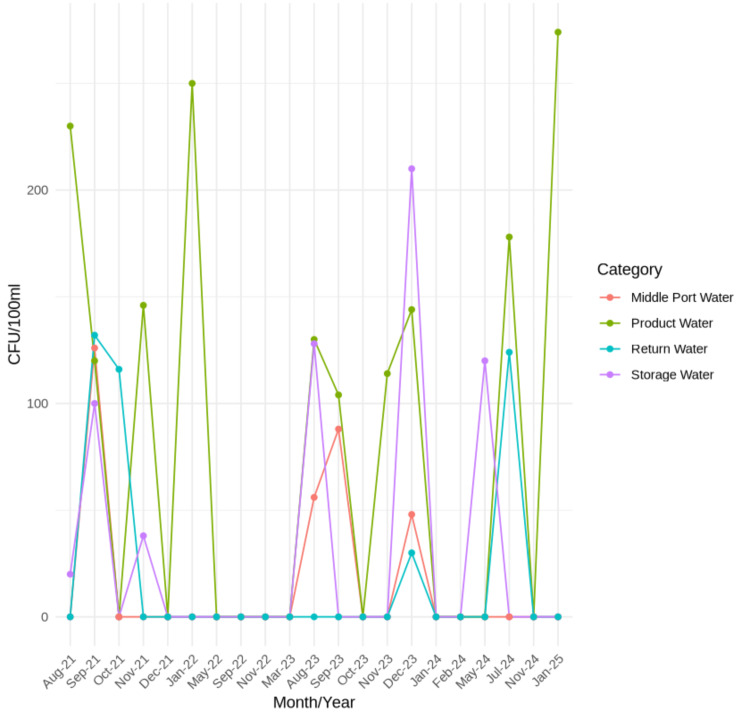
Distribution of four source waters according to number of CFU/100 ml detected

The microbiological assessment of water samples collected from August 2021 to January 2025 demonstrated fluctuating microbial contamination levels across different water sources. Product water exhibited microbial growth in 10 out of 21 samples, with counts ranging from 104 to 274 CFU/100 mL, the highest being in January 2025 (274 CFU/100 mL). Storage water showed microbial presence in five instances, with counts between 20 and 210 CFU/100 mL, the peak occurring in December 2023 (210 CFU/100 mL). Middle port water had microbial contamination in only four samples, with values ranging from 48 to 126 CFU/100 mL. Return water displayed microbial presence in four cases, with counts ranging from 30 to 132 CFU/100 mL, with the highest detected in September 2021 (132 CFU/100 mL) (Figure 1).

**Figure 2:**
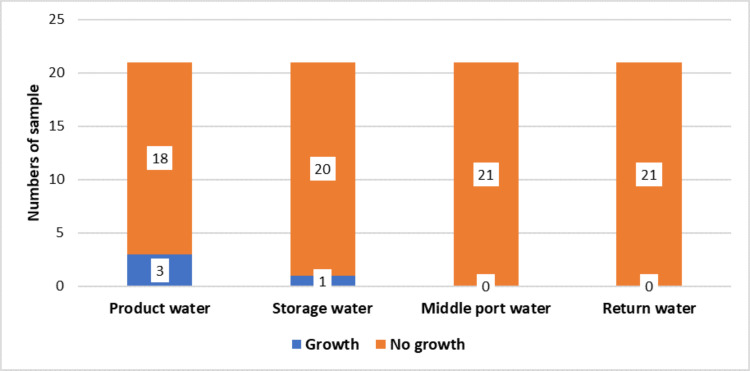
Unsatisfactory growth from different ports according to AAMI

Out of the total 21 samples collected, unsatisfactory microbial growth was observed in three samples of product water and one sample of storage water as per the Association for the Advancement of Medical Instrumentation (AAMI) standard, whereas no unsatisfactory growth was detected in any of the middle port water or return water samples (Figure 2).

The analysis of aerobic bacterial species detected in different water sources using the Vitek 2 Compact (BIOMÉRIEUX, France) revealed varying distributions of microbial growth, as depicted in Table 3. *Acinetobacter baumannii* was the most common organism, as it was present in all four water types.

**Table 3:** Distribution of growth of aerobic bacterial species detected according to source water by Vitek 2 Compact NG: no growth

Aerobic bacterial species	Product water	Storage water	Middle port water	Return water
Acinetobacter baumanii	Positive	Positive	Positive	Positive
Acinetobacter johnsonii	NG	Positive	NG	NG
Acinetobacter lwoffi	NG	NG	NG	Positive
Acinetobacter ursingii	NG	NG	Positive	NG
Aeromonas hydrophila	Positive	NG	NG	NG
Chrysobacterium indologenes	NG	Positive	NG	NG
Comamonas testosteroni	NG	Positive	NG	NG
Cupriavidus pauculus	Positive	NG	NG	NG
Enterobacter aerogenes	NG	Positive	Positive	NG
Klebsiella oxytoca	NG	NG	NG	Positive
Klebsiella pneumoniae	NG	NG	NG	Positive
Pseudomonas chlororaphis	Positive	NG	NG	NG
Ralstonia pickettii	Positive	NG	NG	Positive
Staphylococcus lentus	Positive	NG	NG	NG
Staphylococcus pseudintermedius	Positive	NG	NG	NG
Stenotrophomonas maltophila	NG	NG	Positive	NG

## Discussion

Ensuring the microbiological safety of dialysis water is essential in preventing severe complications such as pyrogenic reactions, sepsis, and chronic inflammation in HD patients. This study assessed the bacteriological quality of treated water and dialysate used in the HD unit of a tertiary care center in Imphal, Manipur.

Our findings align with previous studies, such as the one conducted by Oumokhtar et al. in Morocco, which reported the presence of numerous Gram-negative bacteria in the dialysate and water distribution system. They identified 52.8% of the genus *Pseudomonas *and also detected *Acinetobacter haemolyticus* and *Ralstonia pickettii*, highlighting the potential for biofilm formation within the piping system and dialyzers [12].

The results revealed fluctuations in microbial contamination levels across different water sources, with *Acinetobacter *species being the most frequently detected microorganism. These bacteria can rapidly multiply even in water treated via distillation, deionization, reverse osmosis, and softening. If bacterial contamination exceeds permissible limits, patients are at risk of sepsis or endotoxemia caused by Gram-negative bacteria [15]. Given that dialysis water directly interacts with patient blood via the dialysis membrane, strict microbiological and chemical quality standards have been established.

The AAMI and the European Pharmacopoeia recommend stringent limits, with AAMI setting an upper threshold of 200 CFU/mL for microbial contamination and 2 IU/mL for endotoxins [14]. Our study identified instances where microbial counts exceeded permissible levels, particularly in product water, with CFU counts ranging from 230 to 274 CFU/100 mL. These findings highlight the necessity of continuous monitoring and rigorous adherence to water treatment and maintenance protocols. Similar concerns were reported in a survey conducted in Japan, where 3.9% and 2.6% of dialysis fluid samples exceeded acceptable bacterial contamination limits in 2006 and 2007, respectively [13].

In addition, a study by Asserraji et al. in Saudi Arabia found no contamination in dialysate samples; however, 9.2% of treated water samples had unacceptable bacterial levels. They recommended frequent disinfection of water treatment facilities to maintain high-quality dialysis water [16]. Our study supports this observation, as no contamination exceeding acceptable limits was found in middle port water and return water samples, emphasizing the effectiveness of the hospital’s disinfection protocols.

The detection of multidrug-resistant organisms such as *Acinetobacter baumannii *and *Klebsiella pneumoniae *in return water further underscores the need for stringent infection control measures. *Acinetobacter baumannii*, in particular, has been widely reported in HD settings due to its high antibiotic resistance and ability to form biofilms [17,18]. Its presence across all four water types poses a significant risk to immunocompromised patients. Cross-contamination between dialysis water and the patient’s bloodstream via back-filtration is another potential hazard, necessitating the use of advanced filtration techniques and regular system disinfection. The recent detection of *Aeromonas *species in the most recent sample (January 2025) also raises concerns about periodic lapses in water quality monitoring and potential new contaminants.

This study has several limitations. One significant limitation is the absence of a comparison with other dialysis units in the region, which would have provided a broader understanding of water quality. In addition, endotoxin assays were not conducted due to financial constraints, which limits the assessment of potential endotoxin contamination. The molecular characterization of bacterial isolates was also not performed, restricting insights into the resistance profiles of detected pathogens. Furthermore, data on the disinfection practices employed in the HD unit were not collected, preventing us from assessing their potential impact on water quality. Lastly, due to feasibility constraints and limited resources, this study was conducted at a single center, limiting the generalizability of the findings. Future research should prioritize genomic surveillance of bacterial contaminants, evaluate the effectiveness of various disinfection protocols, and investigate the influence of water quality on clinical outcomes in dialysis patients.

## Conclusions

Our study highlights the variability in microbiological contamination levels in dialysis water and the predominance of *Acinetobacter *species as major contaminant. Given the potential risks associated with microbial contamination in HD units, there is an urgent need to reinforce water treatment protocols, ensure regular monitoring, and implement stringent infection control measures. Adherence to international standards for dialysis water quality can significantly improve patient safety and reduce the risk of dialysis-related infections.
